# West Nile virus host-vector-pathogen interactions in a colonial raptor

**DOI:** 10.1186/s13071-017-2394-z

**Published:** 2017-09-29

**Authors:** Zoltán Soltész, Károly Erdélyi, Tamás Bakonyi, Mónika Barna, Katalin Szentpáli-Gavallér, Szabolcs Solt, Éva Horváth, Péter Palatitz, László Kotymán, Ádám Dán, László Papp, Andrea Harnos, Péter Fehérvári

**Affiliations:** 1 Lendület Ecosystem Services Research Group, MTA Centre for Ecological Research, Vácrátót, Hungary; 2 Hungarian Natural History Museum, Budapest, Hungary; 3 National Food Chain Safety Office, Veterinary Diagnostic Directorate, Budapest, Hungary; 4 Department of Microbiology and Infectious Diseases, University of Veterinary Medicine, Budapest, Hungary; 5 Viral Zoonoses, Emerging and Vector-Borne Infections Group, Institute of Virology, University of Veterinary Medicine, Vienna, Austria; 6 MME/BirdLife Hungary, Red-footed Falcon Conservation Working Group, Budapest, Hungary; 7 Körös-Maros National Park Directorate, Szarvas, Hungary; 8 Hungarian Academy of Sciences, Biological Section, Budapest, Hungary; 9 Department of Biomathematics and Informatics, University of Veterinary Medicine, Budapest, Hungary

**Keywords:** Culicidae, Transmission ecology, Mosquito trap, Arthropod vector, Passive immunity, Host competence, *Falco vespertinus*, Lineage 2, Antibody

## Abstract

**Background:**

Avian host species have different roles in the amplification and maintenance of West Nile virus (WNV), therefore identifying key taxa is vital in understanding WNV epidemics. Here, we present a comprehensive case study conducted on red-footed falcons, where host-vector, vector-virus and host-virus interactions were simultaneously studied to evaluate host species contribution to WNV circulation qualitatively.

**Results:**

Mosquitoes were trapped inside red-footed falcon nest-boxes by a method originally developed for the capture of blackflies and midges. We showed that this approach is also efficient for trapping mosquitoes and that the number of trapped vectors is a function of host attraction. Brood size and nestling age had a positive effect on the number of attracted *Culex pipiens* individuals while the blood-feeding success rate of both dominant *Culex* species (*Culex pipiens* and *Culex modestus*) markedly decreased after the nestlings reached 14 days of age. Using RT-PCR, we showed that WNV was present in these mosquitoes with 4.2% (CI: 0.9–7.5%) prevalence. We did not detect WNV in any of the nestling blood samples. However, a relatively high seroprevalence (25.4% CI: 18.8–33.2%) was detected with an enzyme-linked immunoabsorbent assay (ELISA). Using the ELISA OD ratios as a proxy to antibody titers, we showed that older seropositive nestlings have lower antibody levels than their younger conspecifics and that hatching order negatively influences antibody levels in broods with seropositive nestlings.

**Conclusions:**

Red-footed falcons in the studied system are exposed to a local sylvatic WNV circulation, and the risk of infection is higher for younger nestlings. However, the lack of individuals with viremia and the high WNV seroprevalence, indicate that either host has a very short viremic period or that a large percentage of nestlings in the population receive maternal antibodies. This latter assumption is supported by the age and hatching order dependence of antibody levels found for seropositive nestlings. Considering the temporal pattern in mosquito feeding success, maternal immunity may be effective in protecting progeny against WNV infection despite the short antibody half-life measured in various other species. We conclude that red-footed falcons seem to have low WNV host competence and are unlikely to be effective virus reservoirs in the studied region.

## Background

West Nile virus (WNV) is the most widespread member of the arthropod-borne group of the genus *Flavivirus*, family *Flaviviridae* [1]. Virus strains belonging to genetic lineages 1 and 2 have been causing an increasing number of epidemics in North America [2–4] and Europe [5–12]. Today, WNV is considered one of the most important pathogens causing viral neurological disease in humans [13].

The virus is maintained in an enzootic cycle between vectors and avian hosts, while humans [14], equines [15] and other vertebrate taxa are predominantly dead-end hosts [16]. Therefore, to assess human infection risks and predict the spatio-temporal patterns of disease outbreaks it is vital to better understand the complex avian host-mosquito vector transmission ecology of WNV [17, 18]. A wide array of bird species have been identified as potential virus amplifying hosts [19]. Competent arthropod vectors also belong to a range of taxa [20, 21];, however, ornithophilic mosquitoes (Diptera: Culicidae) are established to be the group predominantly responsible for maintaining the sylvatic cycle of the virus.

Pathogenicity in birds seems to be rather species-specific, and the effect of infection ranges from subclinical to rapid development of fatal neuropathy [22]. WNV can also have a substantial negative impact on an avian population and may even demand attention in the conservation management of high priority species [22, 23]. However, morbidity and mortality rates do not necessarily reflect the epidemiological role of a host species [24]. A more sophisticated approach is to evaluate or quantify host competence, i.e. the ability of a host to generate infection in another susceptible host [25, 26]. Recent studies focusing on WNV host competence were able to pinpoint avian “superspreader” and “supersuppressor” species in North America, and through these, they were able to explain the geographical variation in human spillover rates [26]. In the complex WNV host-vector system, host competence is a function of the magnitude and length of viremia, vector contact rates and host mortality rates [25]. Estimating these parameters for individual species, however, requires a combination of laboratory experiments and field studies which may not be feasible for endangered species. Here, we present a case study where we implemented a comprehensive study design using various methods simultaneously to evaluate host competence of a high conservation value species in WNV circulation under natural conditions.

The studied avian host was the red-footed falcon (*Falco vespertinus*), a species of high international conservation concern [27, 28]. WNV has been reported to cause central nervous disease and mortality in a few sporadic cases for nestlings, but not in adult birds in Hungary [29]. Red-footed falcons are long-range trans-Saharan migrants [30] and may therefore be amongst the candidate species responsible for large spatial scale dispersal of WNV. These raptors are also facultative colonial breeders [31–33] and therefore potentially more vulnerable to rapidly spreading infections [34] compared to territorially breeding birds. Nestlings and juvenile birds are thought to be important in viral amplification [35, 36] as they are localized in the nest and presumably have a less effective immune response against infections. We, therefore, concentrated on red-footed falcon broods and their relationship with vectors and WNV.

Despite its importance, host-vector interaction in WNV transmission ecology studies is often neglected as there are no widely accepted methods currently available to quantify it. Instead, vectors collected with traps that attract mosquitoes through visual and/or olfactory cues (e.g. CDC traps) are used to assess virus presence, quantify virus prevalence in vectors and as a proxy to potential vector loads on hosts [37]. Albeit these methods are cheap and easy to implement they only account for vector abundance, or rather the availability of mosquitoes reacting to the attractant, but completely fail to provide information on actual host-vector contact rates. A more promising method is to use live birds as baits to attract mosquitoes [38]. However, these are limited in the possible number of bait-species that can be used and also fail to account for avoidance strategies of hosts. Tomás et al. [39] suggested and used an easy yet effective method to quantify biting midges (Ceratopogonidae) and blackflies (Simuliidae) in Passerine nest-boxes with the help of a non-invasive adhesive. If also applicable to trap ornithophilic mosquitoes, this method may revolutionize *in situ* WNV transmission ecology studies as it allows to directly measure the vector species composition and the effects of nestling characteristics on attracted and blood-fed vectors.

Here, we initially aimed to evaluate whether WNV vectors can be trapped directly in the vicinity of red-footed falcon broods and whether we can quantify attraction patterns, virus prevalence, and blood-feeding success of vectors attracted by the studied hosts. Simultaneously, we aimed to estimate WNV status of falcon broods through estimating seroprevalence and frequency of nestlings in viremia, to assess the population level effects of WNV on the host species, and to evaluate the potential virus reservoir role of red-footed falcons.

## Methods

### Study area

Field work was carried out during June and July each year from 2010 to 2012, at the Vásárhelyi-plains (46°28'16"N, 20°36'17"E) protected an area of the Körös-Maros National Park Directorate in southern Hungary. The study site holds 4 artificial nest-box colonies where over 100 pairs of red-footed falcons breed each year [40], along with numerous kestrels (*Falco tinnunculus*), jackdaws (*Corvus monedula*) and long-eared owls (*Asio otus*) [41]. Breeding parameters of birds using the nest-boxes was collected within the scope of an ongoing long-term research program [40–43]. Therefore, detailed information such as egg laying dates, clutch size, brood size, and fledging success was readily available. All broods selected for the study were breeding in the same type of standard nest-box. The area is renowned for a large saline lake and other wetlands that are important stop-over and wintering sites of various wader and geese species and common cranes (*Grus grus*). The surrounding habitat is characterized by a mosaic of grasslands and arable fields, with an extensive network of channels and drainage ditches, providing ample possibilities for blood sucking dipterans to breed.

### Mosquito sampling

**Fig. 1:**
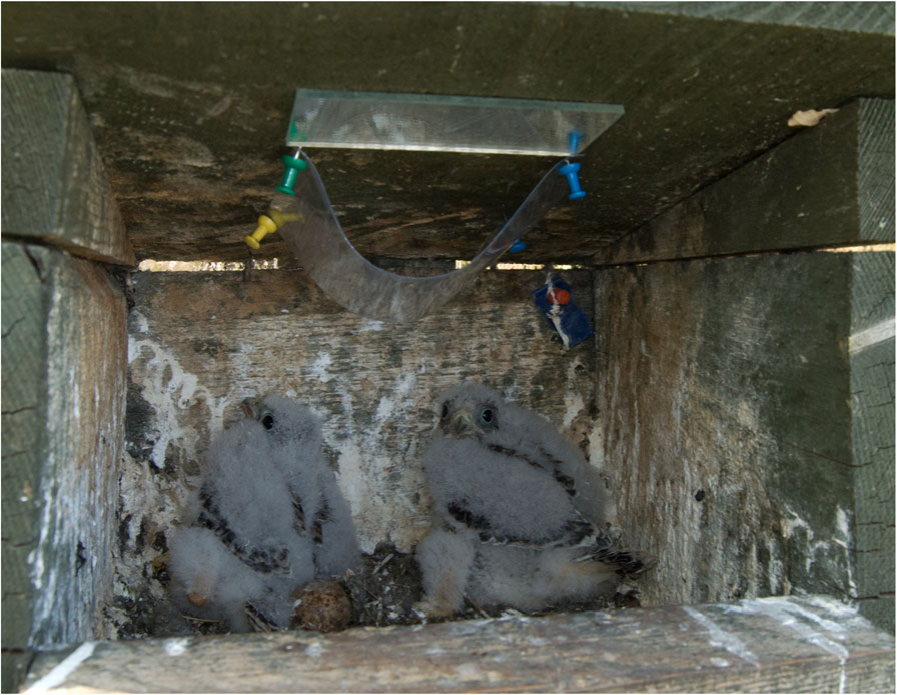
The device used to trap mosquitoes attracted by falcon broods. The upper side of the plastic sheet is covered with an adhesive gel that traps insects landing on the surface. The sheets were left in this position for 24 h

We used a modified version of the methods described by Tomás et al*.* [39] to estimate the number of vectors attracted by red-footed falcon broods. Initially, we applied 5 ml gel adhesive (Johnson’s Baby Oil Gel with Chamomile; Johnson & Johnson, Dusseldorf, Germany) on one side of 10 × 15 cm transparent (0.2 mm) plastic sheets. We then secured these sheets (with the gel facing upwards) on the inner side of the nest-boxes’ roofs for 24 h. The sheets were secured with board pins in a fashion to create an arc, thus allowing ample space for flying arthropods to get trapped (Fig. 1). As opposed to other adhesives, the advantage of the gel is that it is easily dissolved with petrol, leaving trapped arthropods unharmed. This allows trapped Culicidae specimens to be reliably identified, sexed and it is also possible to evaluate their feeding status. All collected animals were stored in 70% ethanol after the gel was dissolved. The species, sex and feeding status (blood-fed or not) was identified under a stereomicroscope (Olympus SZ-50, Olympus Co. Tokyo, Japan).

Sampling was carried out in three consecutive breeding seasons from 2010 to 2012. Each year we first randomly selected empty nest-boxes within each colony. These were used to test whether hematophagous vectors are attracted to either the nest-box or the adhesive gel alone (56 samples collected) by comparing the results obtained here with that in active nests. At the same time, we used a stratified random sampling approach to select broods where mosquito traps were placed. Strata considered were colony and breeding stage, the latter classified into five groups; before hatching (incubation), 1st week, 2nd week, 3rd week and 4th week after hatching. Red-footed falcons incubate throughout the night and also brood young nestlings at night. Presumably, mosquitoes react to the incubating parent rather than the eggs themselves. Thus our analyses may be interpreted as a comparison between a single adult (incubation), an adult with small nestlings (1st week) and older nestlings alone.

Weather is a key factor controlling mosquito activity [44, 45] and consequently WNV infection rates [46]. Therefore, we only selected three days each year for sampling to minimize variation caused by changes in ambient temperature and humidity. On each date broods in various breeding stages were sampled, thus creating a cross-section of the host population. Altogether a total of 309 samples collected in 166 clutches were used in the analyses (Table 1).

**Table 1:** Number of mosquito sampling events in nest-boxes according to year and breeding stage. Control samples derive from boxes where no breeding occurred in the given year. WNV prevalence estimates in mosquitoes and seroprevalence estimates in nestlings was carried out in 2011

Year	Breeding stage						
	Incubation period	1st week	2nd week	3th week	4th week	No. of controls	Total no. of sampling events
2010	1	7	13	15	1	6	37
2011	34	10	16	27	20	19	107
2012	58	34	23	36	14	31	165
total	93	51	52	78	35	56	309

### Assessing WNV prevalence in vectors

WNV prevalence has been shown to cumulate in vertebrate hosts late in the summer [47], therefore to minimize laboratory costs and to maximize the probability of detecting WNV presence we only used mosquitoes trapped in the late breeding stage in 2011. We first grouped the samples by species, sex and according to the nest-box, the animals were trapped in. Specimens that were blood filled were excluded from the analyses to avoid inconclusive results. A total of 779 mosquitoes of three species (*Culex pipiens*, *Culex modestus* and *Coquillettidia richiardii*) were investigated, the majority being *Cx. pipiens* (725 individuals). We then selected every 6th specimen from these subsets for individual WNV identification. The remaining animals were pooled (5 × 4 = 20 individuals/pool) and also analysed [*Cx. pipiens*: 145 individuals, 29 pools; *Cx. modestus*: 3 pools (20 + 20 + 7 individuals); *Cq. richiardii*: 1 pool (7 individuals)]. Samples were tested for the presence of WNV nucleic acid (RNA) by a reverse-transcriptase - polymerase chain reaction (RT-PCR) using the primer pair FL1-f and FL1-r as described earlier [48]. Specific PCR products were sequenced directly from both ends with the same primers [48].

### WNV seroprevalence in red-footed falcon nestlings

We used 42 broods that were also selected for mosquito abundance sampling to assess WNV seroprevalence in the studied population. The samples were chosen to represent both the spatial heterogeneity of breeding pairs (colonies) and temporal heterogeneity of the egg laying dates. Blood samples of 0.8–1.0 ml were taken by basilic venipuncture from fledgelings (*n* = 139) reaching the second half of the breeding stage (17–24 days) in 2011. Sera were separated from coagulated blood samples and stored at -20 °C until processing. The remaining serum and cellular elements were stored at -80 °C until being further processed by simultaneous RNA and DNA extraction using the Roche High Pure Viral Nucleic Acid kit (Lewes, United Kingdom). Serum samples were tested for the presence of anti-WNV antibodies using the ID Screen® West Nile Competition ELISA kit (ID VET, Montpellier, France), according to the manufacturers’ instructions. This diagnostic kit detects IgY antibodies by competitive ELISA directed against the Pr-E envelope protein of the West Nile virus. We used 2 ELISA plates for the analyses. Obtained optical density (OD) values were transformed into OD ratios (i.e. Competition Rate = (OD _sample_ / OD _Negative control_) × 100). These ratios were then handled and analysed with two different approaches. First, we elaborated the recommendations of the manufacturer and defined cut off levels of the test as positive (competition rate ≤ 40%); doubtful (40% < competition rate ≤ 50%); and negative (competition rate > 50%). Secondly, we considered OD ratio as a proxy for antibody levels (e.g. [49, 50]) and analysed these values on a continuous scale (see Statistical analyses). Blood samples were also tested for the presence of WNV nucleic acid (RNA) with the same technique described above.

### Statistical analyses

To understand the relationship between blood-sucking dipteran abundance and host traits we used generalized linear mixed effects models (GLMM) with Poisson distribution and log link function [51–53]. In the next step, we used GLMMs with binomial distribution and logit link function to assess how the ratio of blood filled parasites is affected by these variables. Random factors were the date of sampling, Nest ID and Colony ID for all aforementioned models. All dipteran species were analysed in separate models.

To estimate the WNV seroprevalence in red-footed falcon nestlings, we applied the methods described in Messam et al. [54]. We used the same procedure to estimate WNV prevalence in the vector species, using only the non-pooled samples for the analysis.

We used linear mixed effects (LME) models [51, 55] to estimate the relationship between WNV ELISA OD ratios and nestling characteristics. First, we selected ELISA-positive nestlings and modelled their OD ratios as a function of nestling age measured in days. We then selected all nestlings in broods with at least one WNV-seropositive nestling and assessed their hatching order based on weight (measured with 300 g spring scale to the nearest 2 g), the length of central tail feathers (measured with a ruler to the nearest 1 mm), wing chord (measured with a ruler to the nearest 1 mm) and wing bone (measured with a caliper to the nearest 0.1 mm) lengths recorded at the time of sampling. This hatching order was subsequently used as a predictor for nestling OD ratios. The Nest ID and the ELISA Plate ID were used as random factors in case of both LME models. We also ran the models with the full set of nestlings and obtained numerically similar results.

We used the decrease of deviance and the likelihood ratio test (LR) to select non-significant variables in case of all models described above. All analyses were carried out in R, version 3.2.3 [56] using the following packages; *dplyr* [57], *epiR* [58], *epitools* [59], *nlme* [60], *lme4* [53], *car* [61], *effects* [62] *lmeans* [63].

## Results

### Host-dependent mosquito attraction and blood-feeding success patterns

We trapped a total of 11,592 mosquitoes belonging to 4 species from red-footed falcon nest-boxes, namely *Culex pipiens* Linnaeus, 1758 (*n* = 10,203), *Culex modestus* Ficalbi, 1889 (*n* = 1332), *Coquillettidia richiardii* Ficalbi, 1889 (*n* = 56), *Ochlerotatus dorsalis* (Meigen, 1830) (*n* = 1). All trapped individuals were females. As the latter two species had orders of magnitude lower abundance compared to *Cx. pipiens* and *Cx. modestus* we excluded them from further analyses. We did not trap any mosquitoes in control nest-boxes; however, other arthropods like canopy dwelling or non-parasitic nest substrate feeding species were trapped in small numbers.

**Fig. 2:**
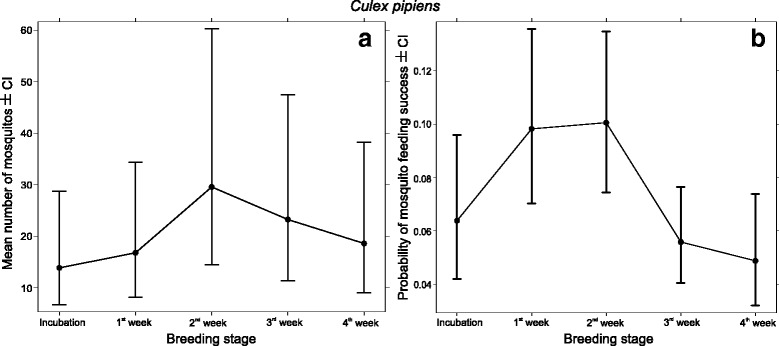
Model estimates (± 95% CI) of the effects of the breeding stage on the number of attracted (**a**) and proportion of blood filled mosquitoes (**b**) of *Cx. pipiens* individuals (see also Tables 2, 3). The values presented here were transformed for the corresponding response scale and calculated for mean brood size. The highest number of attracted ​mosquitoes was in the second week after hatching, and this also coincided with the peak of blood-feeding success

**Fig. 3:**
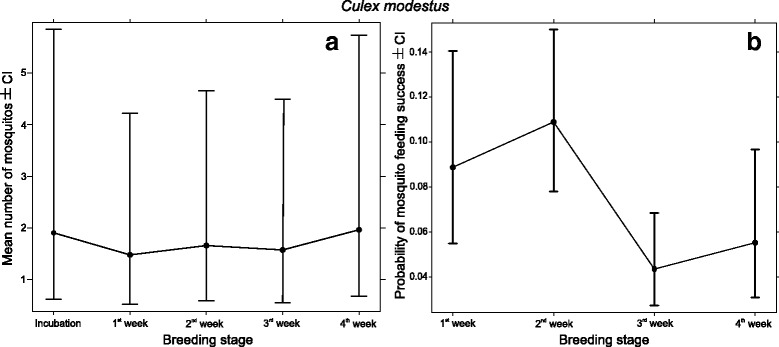
Model estimates (± 95% CI) of the effects of the nestling age on the number of attracted (**a**) and proportion of blood filled mosquitoes (**b**) of *Cx. modestus* individuals (see also Tables 2,3). The values presented here were transformed for the corresponding response scale and calculated for mean brood size. Nestling age did not have a significant effect on the total number of attracted mosquitoes. However, blood-feeding success patterns peaked in the second week after hatching

Brood size and the breeding stage had a significant effect (Poisson GLMM, LR *χ* ^2^ test; brood size *χ* ^2^ = 132.06, *df* = 1, *P* < 0.001, breeding stage *χ* ^2^ = 181.39, *df* = 1, *P* < 0.001) on the number of *Cx. pipens* in nest-boxes. Considering the latter variable, the number of individuals significantly increased after hatching, peaked at the 2nd week, and from here on it significantly decreased until fledging. (Table 2, Fig. 2a). Meanwhile, the ratio of blood-fed *Cx. pipiens* individuals was also significantly affected by breeding stage (Binomial GLMM, LR *χ* ^2^ test, breeding stage *χ* ^2^ = 29.83, *df* = 1, *P* < 0.001; brood size *χ* ^2^ = 0.05, *df* = 1, *P* = 0.82); the probability of finding a blood-engorged mosquito was highest in the 1st and 2nd week after hatching, and later significantly decreased for the second half of the nestling stage (Table 3, Fig. 2b). In the case of *Cx. modestus*, brood size significantly increased while breeding stage did not affect (Poisson GLMM, LR *χ* ^2^ test; brood size *χ* ^2^ = 24.6, *df* = 1, *P* < 0.001; breeding stage *χ* ^2^ = 2.17, *df* = 1, *P* = 0.71) the number of attracted ​mosquitoes (Table 2, Fig. 3a). The pattern of the ratio of blood-fed *Cx. modestus* (Fig. 3b) individuals resemble the pattern of *Cx. pipiens* (Fig. 2b). However, the data did not allow to completely replicate the same analysis as only 2 blood-fed *Cx. modestus* individuals were trapped in the incubation period (Table 3, Fig. 3b).

**Table 2:** Comparisons of the effect of breeding stage on overall mosquito abundance using consecutive contrasts for Poisson GLMMs. The reported values show the mean differences in abundance between the corresponding breeding stage pairs (see also Figs. 2a, 3a)

Species	Variable	Contrast levels	Difference in abundance	SE	*P*-value
*Cx. pipiens*	Breeding stage	1st week *vs* incubation	2.92	1.8	0.106
		2nd week *vs* 1st week	12.76	4.75	0.007
		3rd week *vs* 2nd week	-6.31	2.55	0.013
		4th week *vs* 3rd week	-4.64	1.98	0.02
*Cx. modestus*	Breeding stage	1st week *vs* incubation	-0.25	0.322	0.43
		2nd week *vs* 1st week	0.11	0.21	0.58
		3rd week *vs* 2nd week	-0.05	0.18	0.77
		4th week *vs* 3rd week	0.22	0.19	0.25

*Abbreviation*: *SE* standard error

**Table 3:** Comparisons of the effect of breeding stage on overall mosquito blood-feeding success using consecutive contrasts for Binomial GLMMs. The reported values show the differences in mean probabilities of blood-feeding success between the corresponding breeding stage pairs (see also Figs. 2b, 3b)

Species	Variable	Contrast levels	Difference in blood-feeding success	SE	*P*-value
*Cx. pipiens*	Breeding stage	1st week *vs* incubation	0.034	0.01	0.031
		2nd week *vs* 1st week	0.002	0.01	0.86
		3rd week *vs* 2nd week	-0.044	0.01	< 0.001
		4th week *vs* 3rd week	0.007	0.01	0.447
*Cx. modestus* ^a^	Breeding stage	2nd week *vs* 1st week	0.021	0.02	0.48
		3rd week *vs* 2nd week	-0.065	0.02	0.001
		4th week *vs* 3rd week	0.011	0.02	0.53

^a^blood-feeding success was not estimated during incubation due to sample size constraints

*Abbreviation*: *SE* standard error

### WNV prevalence in vectors

We found 1 pooled and 6 individual *Cx. pipiens* (*n* = 145) samples to be WNV-positive, corresponding to a 4.2% (95% CI: 0.9–7.5%) virus prevalence assuming perfect test detection (calculated only for individual samples). The positive samples were found in three nest-boxes, each in a different colony, indicating WNV carrying mosquito presence throughout the study site. One nest-box had 5 WNV-positive individual *Cx. pipiens* samples while the remaining 1 individual and 1 pooled sample were from different nest-boxes and different colonies. Sequence analysis determined that the detected virus belongs to the genetic lineage 2 of WNV and it is closely related to the WNV isolates from the study year and previous years [8].

### WNV seropositivity in red-footed falcons

**Fig. 4:**
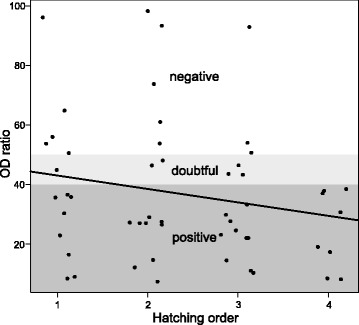
ELISA OD ratios according to hatching order in broods where at least one ELISA-seropositive nestling was found. The black line shows a significant mean decrease in OD ratios (i.e. increase in antibody levels) according to LME model parameter estimate. The grey areas along the y axis depict ELISA classification categories as recommended by the manufacturer. Note that the OD ratio values were jittered along the x-axis. The results show that nestlings that hatched later (i.e. closer to the time of sampling) may have higher antibody levels

Our results showed relatively high seroprevalence among red-footed falcon nestlings; 35 of the sampled 134 individuals were ELISA-positive, while 10 were classified as doubtful (25.4% ± 3.7% SE, CI: 18.8–33.2%). However, we did not detect WNV by RT-PCR in any of the nestling blood samples, suggesting that none of the individuals were viraemic at the time of sampling. Of the 42 broods, 16 had at least one seropositive nestling. We found no evidence of large scale spatial pattern of the observed seropositivity, as the ratio of seropositive broods did not differ significantly between colonies (Fischer’s exact test: *P* = 0.94). Moreover, we could not detect any temporal pattern either, as there was no significant difference in mean egg laying date between broods without seropositive and broods with at least one seropositive nestling (Welch t-test: *t* = -0.1095, *df* = 34.601, *P* = 0.91). However, the OD ratio significantly decreased with increasing nestling age among ELISA-positive nestlings (LME LR *χ* ^2^ test; nestling age *χ* ^2^ = 4.97, *df* = 1, *P* = 0.02) indicating that older seropositive nestlings have lower antibody levels. We also found a significant decrease in OD ratios corresponding with within brood hatching order among broods with at least a single ELISA-positive nestling (LME LR *χ* ^2^ test; hatching order *χ* ^2^ = 5.36, *df* = 1, *P* = 0.02) (Fig. 4). This shows that nestlings hatching later in a brood had higher antibody levels.

## Discussion

Here, we comprehensively investigated vector attraction patterns, blood-feeding success rate, WNV prevalence and host serum seroprevalence under natural conditions in a colonial raptor. Initially, we verified that our modified version of the trap described by Tomás et al. [39] was effective in collecting Culicidae species and that the trapped individuals were attracted by the hosts. The two most common mosquito species (*Cx. pipens* and *Cx. modestus*) attracted by red-footed falcon broods are well known WNV vectors [38, 64]. The number of these vector individuals showed a positive linear relationship with the number of nestlings in a brood, indicating that each nestling may receive similar vector loads regardless of brood size. Mosquitoes use multiple olfactory cues and skin emanations to locate vertebrate hosts [65]. Presumably, larger broods produce increased host stimuli attracting the insects from a larger area, hence the observed pattern.

We also demonstrated nestling age dependent vector attraction and blood-feeding success rate. First, our results indicated that *Cx. pipiens* is disproportionately attracted to nestling age categories where the adult birds are absent compared to age categories where adult presence is presumed (incubation and the 1st week after hatching). It is possible that albeit an incubating adult is larger than the nestlings, a single bird with a complete plumage may be emitting less intensive cues compared to a brood of semi-grown nestlings Secondly, blood-feeding success rates are considerably higher for both *Culex* species in the first two weeks after hatching, and subsequently drop in later breeding stages. This decrease in both attraction and blood-feeding success coincides with the development of body and flight feathers. The gradual shift from downy feathers to juvenile plumage presumably decreases the body surface where mosquitoes may feed [66] and by acting as a better insulator may also decrease host attractiveness. Furthermore, it may also be adaptive for ​mosquitoes to select for young nestlings as the probability of successful blood-feeding may be higher compared to that on fledglings.

It has to be emphasized that our method to quantify mosquito attraction and blood-feeding patterns hinder the estimation of true host-vector contact rates. Nonetheless, it allows us to speculate that younger red-footed falcon nestlings are at higher risk of infection by vector-borne pathogens.

We also showed that the very mosquitoes attracted to the nests harbour WNV. Although the virus was only present in *Cx. pipiens*, this is likely due to the fact that this species was an order of magnitude more abundant than *Cx. modestus*. The obtained prevalence estimate (~4%, 95% CI: 0.9–7.5%) is in the range of that found in North America for Lineage 1 strains [9, 67–70] but somewhat higher than estimated for Hungary in general [71] and the Czech Republic [12]. However, this estimate does not indicate large scale amplification of the virus, despite the fact that the attributes of the studied host-vector system (coloniality, a large number of potential vectors and hosts) would, in theory, allow for effective virus circulation and accumulation. Nonetheless, it is likely that this prevalence estimate indicates a stable WNV sylvatic cycle at the study site.

Although we confirmed WNV presence in vectors directly attracted by red-footed falcon nestlings, the virus was not present in detectable amounts in blood samples of the exposed birds. Despite the lack of viremia, we found that a considerable proportion (~25%) of these nestlings was WNV-seropositive. These seemingly contradicting observations may arise if either all seropositive nestlings were infected soon after hatching and/or the duration of detectable viremia was remarkably short. This scenario is however unlikely based on estimates in viremia magnitude and length of large falcons [72] and a comparative study on multiple species [73]. More probable is that the majority, if not all, seropositive nestlings had maternally derived antibodies against WNV [37, 74]. This is also corroborated by two additional results. First, age dependent increase in ELISA OD ratios, indicate that Ig levels decay with the days elapsed from hatching as opposed to increasing and/or stagnation in infected birds [72]. Secondly, ELISA OD ratios decreased with hatching order, showing that younger nestlings have higher Ig values in nests with at least one seropositive nestling. Red-footed falcons typically hatch 1–2 days apart in a clutch, therefore, both results show that the time-scale of maternally derived antibody decay can be measured in days [75–77]. However, antibody decrease in free ranging birds with the active immune response is detectable over months [78]. Nemeth et al. [79] argued that the rapid decay of maternal antibodies in nestling house sparrows is unlikely to offer effective protection to offspring [79].

If successful blood-feeding probability is nestling age-dependent, it is possible that the efficiency of passive immunity is not exclusively linked to the half-life of maternal antibodies.

## Conclusions

Understanding vector-borne arboviral infection systems in avian hosts requires a comprehensive approach that entails studying host-vector and vector-pathogen interactions. Using a specific trap placed in the vicinity of the brood, culicid vectors directly attracted by the studied hosts can be quantified in natural conditions, and these samples can also be used to estimate viral loads of vectors. WNV has an established sylvatic cycle and poses a direct threat to red-footed falcon nestlings. The array of soft evidence presented here points to the prediction that either nestlings have very short viremia or that a large proportion of red-footed falcon females breeding in the studied population allocates WNV antibodies to their eggs. This also leads to the prediction that the breeding adults have been infected by the virus either at the breeding site or during their wintering period in Africa. Considering the host-vector contact rate patterns, yolk transfer of antibodies may be an efficient measure to protect nestlings with naive immune systems from the clinical effects of a WNV infection. The highest risk of contact with a potential WNV vector is in the first two weeks after hatching when maternal antibodies are expected to be sufficiently high based on estimated decay patterns in other birds. In any case, our results do not allow us to explicitly rule out either of the following two competing theories, i.e. that the observed patterns are either caused by very short viremia or by maternal transfer of antibodies. However, red-footed falcon nestlings are likely to have low WNV host competence under both scenarios and are less likely to be key hosts maintaining the sylvatic cycle of the virus, at least during their breeding season.

## Abbreviations

CI: Confidence interval
ELISA: Enzyme-linked immunoabsorbent assay
GLMM: Generalized linear mixed effects models
LME: Linear mixed effects
SE: Standard error
WNV: West Nile virus
